# Studies on the Alkaloids of the Bark of *Magnolia officinalis*: Isolation and On-line Analysis by HPLC-ESI-MS^n^

**DOI:** 10.3390/molecules18077739

**Published:** 2013-07-03

**Authors:** Renyi Yan, Weihao Wang, Jian Guo, Hongliang Liu, Jianyong Zhang, Bin Yang

**Affiliations:** Institute of Chinese Materia Medica, China Academy of Chinese Medical Sciences, Beijing 100700, China; E-Mails: yanry2009@163.com (R.Y.); olive_wh@126.com (W.W.); guojian555@126.com (J.G.); knox_liu@126.com (H.L.); riskeen@sina.com (J.Z.)

**Keywords:** *Magnolia officinalis*, alkaloid, HPLC-MS^n^, 4-keto-magnoflorine, 3,4-dehydromagnocurarine

## Abstract

The bark of *Magnolia officinalis* is a well-known Traditional Chinese Medicine. In the present study, two new alkaloids, named (*S*)-4-keto-magnoflorine (**6**) and (*R*)-3,4-dehydromagnocurarine (**11**), together with seven known alkaloids: (*S*)-magnoflorine (**5**), *trans*/*cis N*-feruloylputrescine (**7**/**8**), (*R*)-magnocurarine (**10**), (*S*)-tembetarine (**12**), (*R*)-oblongine (**14**), and (*R*)-asimilobine (**17**) were isolated and their structures elucidated by spectroscopic methods, including 1D, 2D NMR, and HRESI-MS. The absolute configurations of the isoquinoline alkaloids **5**, **6**, **10**–**12**, **14**, and **17** were determined by CD. *In vitro* inhibitory activities against aldose reductase, lipase, *α*-glucosidase, DPP–IV and three cancer cell lines (A549, Bel-7402, and HCT-8) were evaluated for all isolated compounds. However, all compounds showed weak activities in all tests at the same concentration as the positive control drugs. An HPLC-ESI-MS^n^ method has been established for screening of alkaloids in the bark of *M*. *officinalis*. A total of 23 alkaloids were identified or tentatively characterized; including 13 aporphines, eight benzylisoquinolines and two amides. Plausible fragmentation pathways of the representative compounds **6**, **7**/**8**, **11**, and **17** were proposed in the present study.

## 1. Introduction

**Figure 1:**
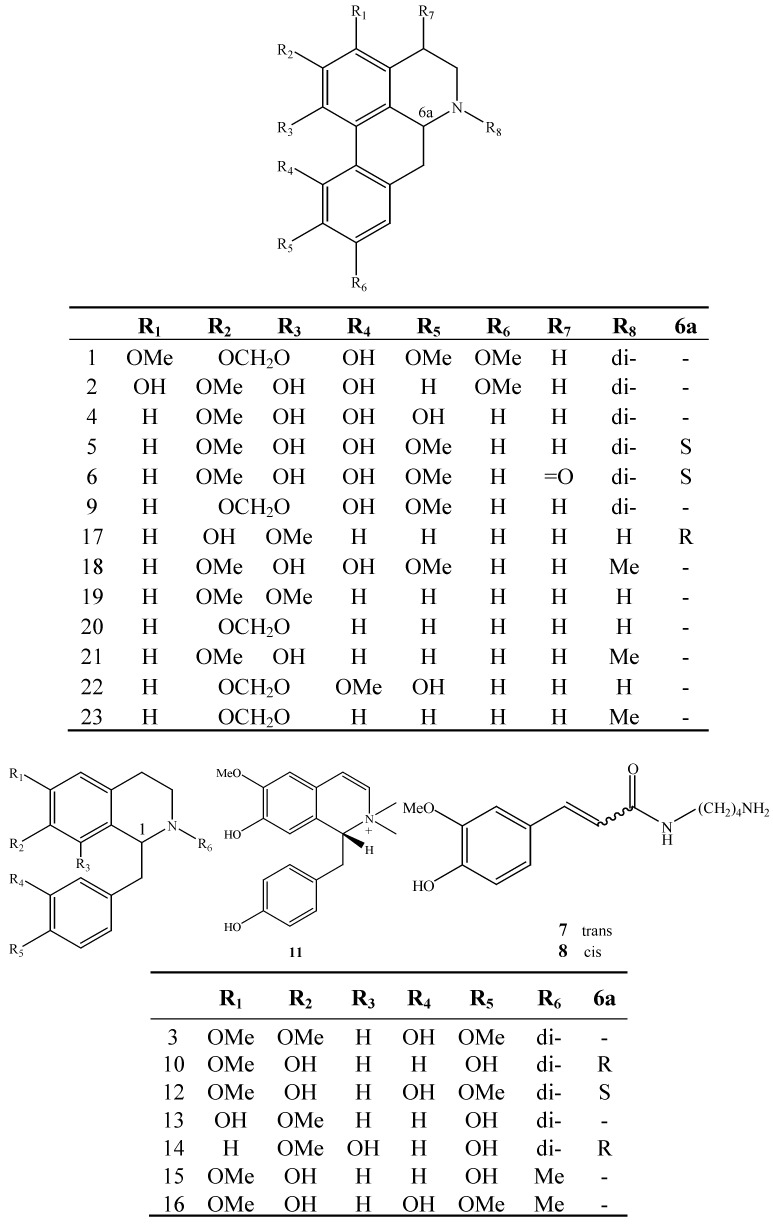
Structures of compounds **1–23**.

The bark of *Magnolia officinalis* (Magnoliaceae), called Houpu in Chinese, has a very wide range of applications. It’s a common ingredient in the treatment of abdominal swelling of various causes and edema. Many of the prescriptions containing Houpu are also aimed at treatment of lung disorders (including cough and asthma) or intestinal disorders [1,2]. Besides the well-known lignans magnolol and honokiol being the active compounds of *M. officinalis*, alkaloids are a group of interesting secondary metabolites of this species, which produces mainly isoquinoline-type alkaloids, the majority of which are aporphine and benzylisoquinoline derivatives [3,4,5,6]. In a previous paper from our group, 11 polar compounds including one major alkaloid—magnoflorine—have been isolated from the stem bark of *M*. *officinalis* [7]. In connection with our interest in the alkaloids of this plant, a further chemical study focusing on an alkaloids-enriched fraction has been conducted. The strategy of this study includes two steps: (1) isolation of main components and complete structural characterization by NMR, MS, and CD spectroscopies. (2) on-line identification of alkaloids by comparing retention time and MS^n^ data. Twenty three alkaloids were identified or tentatively characterized, including 13 aporphines, eight benzylisoquinolines and two amides. Two new alkaloids, named (*S*)-4-keto-magnoflorine (**6**) and (*R*)-3,4-dehydromagnocurarine (**11**), together with seven known alkaloids were isolated (Figure 1).

## 2. Results and Discussion

### 2.1. Structure Elucidation of the Purified Compounds

Compound **6** was obtained as a white amorphous powder. The molecular formula was determined to be C_20_H_22_O_5_N from its HRESI-MS (*m*/*z* 356.1514 [M]^+^; calcd for C_20_H_22_O_5_N, 356.1498) and NMR data. The ^1^H-NMR spectrum showed two adjacent aromatic protons at *δ*_H_ 6.55 (1H, d, *J* = 8.0 Hz) and 6.40 (1H, d, *J* = 8.0 Hz), and one isolated aromatic proton at *δ*_H_ 6.87 (1H, s), which were ascribed to the hydrogens of A and D ring of the aporphine nucleus. Singlets at *δ*_H_ 3.75 (3H, s) and 3.70 (3H, s) attributed to methoxy groups. The signals at *δ*_H_ 3.31 (3H, s) and 2.82 (3H, s) indicated the presence of two *N*,*N*-dimethyl groups in the molecule.

**Figure 2:**
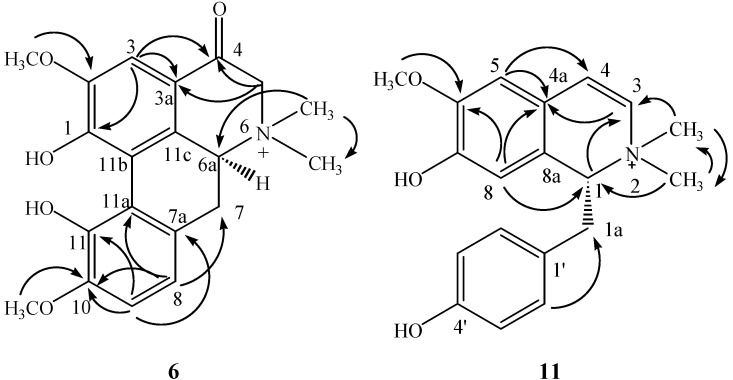
Key HMBC correlations of compounds **6** and **11**.

The ^13^C-NMR data together with a HSQC experiment indicated the presence of 20 carbons in **6**, comprising one ketone carbon at *δ*_C_ 187.4, 12 aromatic carbons between *δ*_C_ 165.2 and 107.2, four methyl groups at *δ*_C_ 58.1, 58.0, 56.2, and 48.0, two methylenes at *δ*_C_ 69.9 and 31.5, and one methine at *δ*_C_ 71.4. The NMR data were similar to those of magnoflorine [8], except for the ketone carbon signal and the low field hydrogen signal at *δ*_H_ 3.91 (2H, s). The long-range ^1^H-^13^C correlation observed between H-3 (δ_H_ 6.87, s) and *δ*_C_ 187.4 confirmed the substitution at C-4 (Figure 2). The NOE correlations between H-3 and *δ*_H_ 3.70 (3H, s), between H-9 and *δ*_H_ 3.75 (3H, s) confirmed the location of methoxys. The absolute configuration of C-6a was established as *S* by the circular dichroism (CD) spectrum, which showed a positive Cotton effect at 235 nm [9]. Thus, the structure of 6 was elucidated as (*S*)-4-keto-magnoflorine.

Compound **11** was obtained as a white amorphous powder. Its molecular formula was determined as C_19_H_22_O_3_N by HRESI-MS (*m/z* 312.1610 [M]^+^ calcd for C_19_H_22_O_3_N, 312.1599). The ^1^H-NMR spectrum showed eight olefinic protons, with one AA'BB' system (δ_H_ 6.68 and 6.71, both d, *J* = 8.8 Hz), two singlets at *δ*_H_ 7.07 and 5.90, and two broad signals at *δ*_H_ 6.91 and 6.21. Other signals were attributed to three methyl groups at *δ*_H_ 3.87 (3H, s), 3.52 (3H, s), and 3.22 (3H, s), and an AB_2_ system at *δ*_H_ 4.60 (dd, *J* = 11.2, 4.8 Hz), 3.43 (dd, *J* = 12.4, 4.8 Hz), and 2.92 (dd, *J* = 12.4, 11.2 Hz). On the basis of the presence of AA'BB' and AB_2_ systems, the basic structure of the molecule was suggested as that of a benzylisoquinoline. In HMBC spectrum, *δ*_H_ 3.52 (3H, s) and 3.22 (3H, s) were correlated with each other, which indicated the both methyl groups were connected to the same hetero atom. In addition, these two methyl groups were also correlated with *δ*_C_ 76.1 and 131.0 which unveiled the presence of a quaternary amine in its structure (Figure 2).

Compared with magnocurarine, the NMR spectral data of **11** showed some differences, including the absence of two aliphatic carbon signals and the presence of two more olefinic carbons at *δ*_C_ 131.0 and 127.6, all of which implied that **11** possessed one more olefinic bond. The location of the olefinic bond at C-3 and C-4 was inferred by the HMBC correlations of H-5/C-4, N-methyl/C-3, and H-1/C-3. Moreover, the NOE correlation between H-5 and *δ*_H_ 3.87 (3H, s) confirmed the location of methoxy. The absolute configuration of C-1 was established as *R* by CD spectrum, which showed a negative Cotton effect at 230 nm [10]. Thus, the structure of **11** was elucidated as (*R*)-3,4-dehydromagnocurarine.

Seven known compounds, namely (*S*)-magnoflorine (**5**) [8], *trans*/*cis N*-feruloylputrescine (**7/8**) [11], (*R*)-magnocurarine (**10**) [5], (*S*)-tembetarine (**12**) [12], (*S*)-oblongine (**14**) [12], and (*R*)-asimilobine (**17**) [13] were isolated and their structures were determined by comparing their spectroscopic data with those reported in the literature. The absolute configuration of known compounds **5**, **10**, **12**, **14**, and **17** was determined by their CD spectra.

### 2.2. Bioactivity

Inhibitory activities against aldose reductase (10 µM), lipase (10 µM), *α*-glucosidase (40 µM), and DPP–IV (10 µM) were evaluated for all isolated compounds **5–8**, **10–12**, **14**, and **17**. All tested compounds showed weak activities at the same concentration as the positive control drugs (epalrestat, orlistat, acarbose, and INDP-2). The inhibition rates of all tested compounds are all less than 20%. In the cytotoxicity bioassay, isolated compounds (5 µg/mL) showed weak activity against the A549, Bel-7402, and HCT-8 cancer cell lines, while the positive control 5-fluorouracil showed an IC_50_ value of 0.40 μg/mL. 

### 2.3. HPLC-ESI-MS^n^ Analysis of Alkaloids in M. officinalis

**Figure 3:**
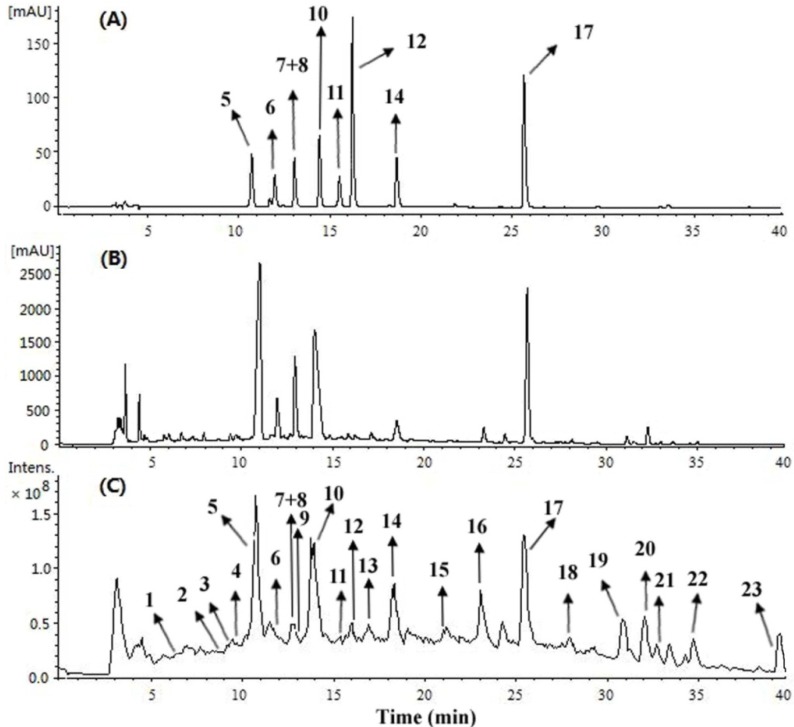
LC-UV/MS chromatograms for the alkaloid standards and the alkaloids-enriched extract of *M. officinalis*. (**A**) UV chromatogram at 283 nm of the nine alkaloid standards; (**B**) UV chromatogram at 283 nm and (**C**) MS TIC chromatogram of the alkaloids-enriched extract. Peak numbers were consistent with those shown in Table 1.

A total of 23 alkaloids were identified or tentatively characterized (Figure 1 and Table 1). Comparing the retention times and MS^n^ spectra with those of the corresponding standards, peaks **5–8**, **10–12**, **14**, and **17** could be unambiguously assigned as (*S*)-magnoflorine (**5**), (*S*)-4-keto-magnoflorine (**6**), *trans*/*cis N*-feruloylputrescine (**7/8**), (*R*)-magnocurarine (**10**), (*R*)-3,4-dehydromagnocurarine (**11**), (*S*)-tembetarine (**12**), (*S*)-oblongine (**14**), and (*R*)-asimilobine (**17**), respectively (Figure 3). For other alkaloids, the structures were tentatively identified based on their MS fragmentation behavior (Table 1). Accordingly, compounds **1–2**, **4–6**, **9**, and **17–23** were ascribed to aporphine alkaloids, compounds **3**, **10**, and **11–16** were ascribed to benzylisoquinoline alkaloids. As well known, the two major types of alkaloids which have been found in *M*. *officinalis* are the aporphine and benzylisoquinoline ones.

**Scheme 1:**
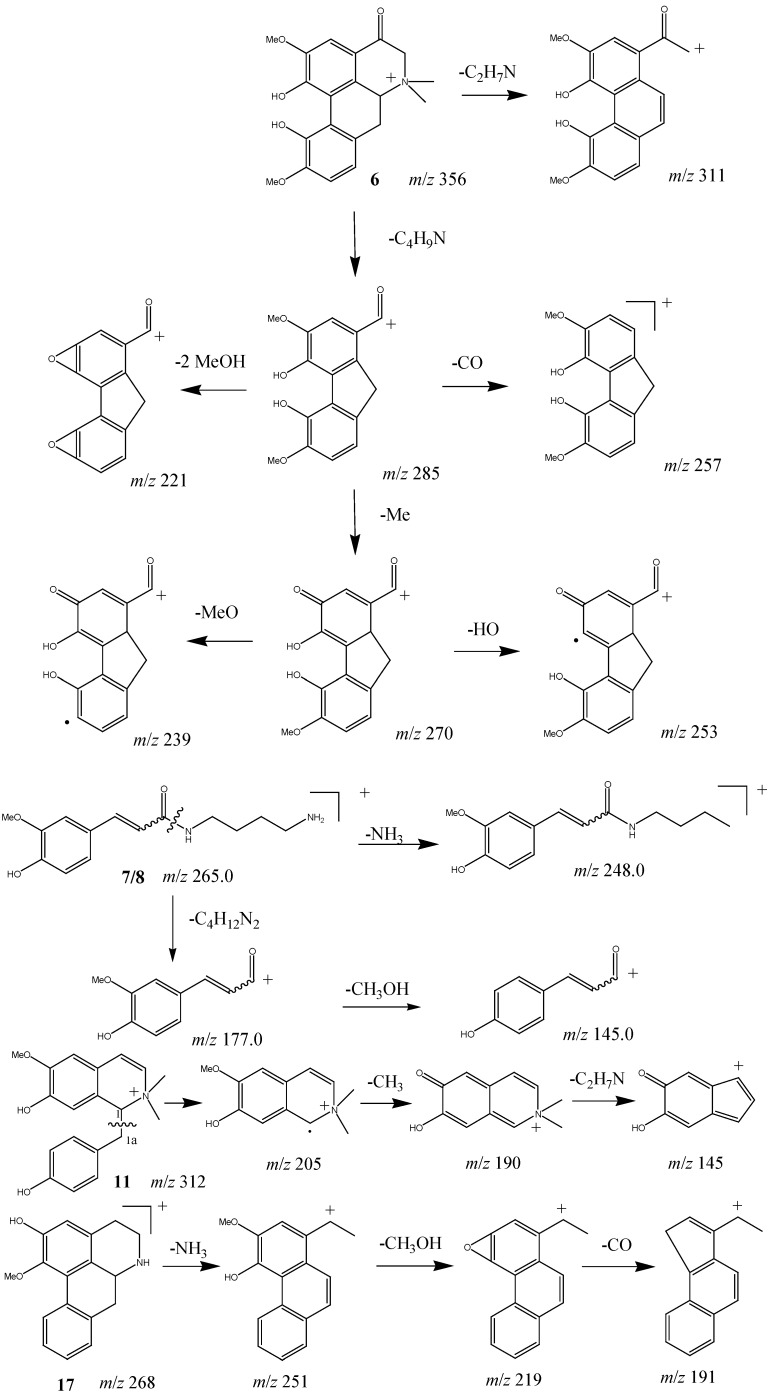
Proposed mechanistic pathway for the fragmentations of alkaloids **6**, **7/8**, **11**, and **17**.

Positive ion mode of ESI was selected in the present study. In general, quaternary alkaloids gave [M]^+^ ions, tertiary and secondary alkaloids gave [M+H]^+^ ions (Table 1). Isolated standards **5** and **17** generated significant [M–(CH_3_)_2_NH)]^+^ or [M+H–NH_3_]^+^ fragment ions as the base peak in their respective MS^2^ spectra, corresponding to quaternary and secondary aporphines, respectively [14]. Based on the mentioned characteristics, compounds **1**, **2**, **4**, and **9** should be quaternary aporphines due to the significant [M–(CH_3_)_2_NH]^+^ fragment ions in their MS^2^ spectra. Similarly, compounds **19**, **20**, and **22** should be secondary aporphines due to the significant [M+H–NH_3_]^+^ fragment ions in their MS^2^ spectra. The MS^2^ fragmentation of compounds **18**, **21**, and **23** provided significant [M+H–CH_3_NH_2_]^+^ ions in the product-ion spectra, suggesting one methyl group at the nitrogen atom of these compounds. The benzylisoquinoline alkaloids **10** and **12–16** showed obvious cleavages between C-1 and C-1a to produce the significant ion fragments at *m*/*z* 107 or 137, which were ascribed to hydroxybenzyl and 3-methoxy-4-hydroxybenzyl fragments, respectively [15]. However, isolated benzylisoquinoline alkaloid **11** gave an ion at *m*/*z* 205 as the base peak in the MS^2^ spectrum, undergoing the loss of a hydroxybenzyl fragment. Similarly, an ion at *m*/*z* 221 in MS^2^ spectra of compound **3** indicated the substituent type of ring C. In the MS^2^ spectra, compounds **3**, **10** and **12–14** showed a characteristic [M-(CH_3_)_2_NH]^+^ loss, whereas compounds **15** and **16** showed a characteristic [M−CH_3_NH_2_]^+^ loss indicating the number of methyl groups at the nitrogen atom. The ion peaks derived from loss of CH_3_OH, CH_3_O, H_2_O, CH_3_, CO and CH_2_O were characteristic of aporphine and benzylisoquinoline alkaloids, and served for their structural elucidation [15]. A plausible fragmentation pathway of the representative compounds **11** and **17** is shown in Scheme 1.

**Table 1:** Retention time and MS data of alkaloids in stem bark of *M*. *officinalis.*

**Peak no.**	***t*_R_ (min)**	**Proposed compounds**	**MS**	**ESI-MS^n^*m*/*z* (% base peak)**
**1**	6.3	*N*-Methylocoxylonine (**1**)	400 [M]^+^	MS^2^ [400]: 369 (10), 355 (100), 337 (15), 323 (95); MS^3^ [355]: 340 (5), 323 (100), 309 (7); MS^3^ [323]: 295 (100)
**2**	8.7	1,3,11-Trihydroxy-2,9-dimethoxy-6,6-dimethylaporphinium (**2**)	358 [M]^+^	MS^2^ [358]: 340 (20), 313 (100), 295 (80), 281 (75); MS^3^ [313]: 298 (20), 285 (65), 281(90), 253 (100)
**3**	9.3	*N*-Methyllaudanidinium (**3**)	358 [M]^+^	MS^2^ [358]: 340 (11), 313 (100), 295 (60), 221 (54); MS^3^ [313]: 295 (20), 285 (99), 281 (100), 253 (95)
**4**	9.9	1,9,10-Trihydroxy-2-methoxy-6,6-dimethylaporphinium (**4**)	328 [M]^+^	MS^2^ [328]: 283 (98), 265(90), 257 (100)
**5**	10.8	(*S*)-Magnoflorine (**5**)	342 [M]^+^	MS^2^ [342]: 311 (20), 297 (100), 265 (53); MS^3^ [297]: 282 (13), 264 (100), 237 (12)
**6**	11.8	(*S*)-4-Keto-magnoflorine (**6**)	356 [M]^+^	MS^2^ [356]: 311.0 (5), 285 (100); MS^3^ [285]: 270 (60), 257 (83), 253 (80), 239 (78), 221 (100)
**7/8**	12.8	*trans*/*cis N*-Feruloylputrescine (**7/8**)	265 [M+H]^+^	MS^2^ [265]: 248 (75), 177 (100); MS^3^ [177]: 145 (100)
**9**	12.9	*N-*Methylbulbocapnine (**9**)	340 [M]^+^	MS^2^ [340]: 309(5), 295 (100), 203 (17); MS^3^ [295]: 263 (100)
**10**	14.0	(*R*)-Magnocurarine (**10**)	314 [M]^+^	MS^2^ [314]: 269 (100), 175 (39), 107 (66); MS^3^ [269]: 237 (24), 175 (55), 107 (100)
**11**	15.5	(*R*)-3,4-Dehydromagnocurarine (**11**)	312 [M]^+^	MS^2^ [312]: 205 (100), 190 (20), 145 (11); MS^3^ [205]: 190.0 (100), 145 (15);
**12**	16.2	(*S*)-Tembetarine (**12**)	344 [M]^+^	MS^2^ [344]: 299 (99), 175 (100), 137 (83); MS^3^ [175]: 143 (100)
**13**	16.9	Lotusine (**13**)	314 [M]^+^	MS^2^ [314]: 269 (23), 237 (27), 205 (35), 175 (48),107 (100); MS^3^ [269]: 175 (99), 107 (100)
**14**	18.4	(*R*)-Oblongine (**14**)	314 [M]^+^	MS^2^ [314]: 269 (51), 254 (8), 237 (11), 175 (13), 137 (32), 107 (100)
**15**	21.7	*N*-Methylcoclaurine (**15**)	300 [M+H]^+^	MS^2^ [300]: 269 (100), 237 (10), 192 (15), 107 (50); MS^3^ [269]: 237 (20), 175 (70), 107 (100)
**16**	23.3	Reticuline (**16**)	330 [M+H]^+^	MS^2^ [330]: 299 (5), 192 (100), 177 (3), 137 (4)
**17**	25.4	(*R*)-Asimilobine (**17**)	268 [M+H]^+^	MS^2^ [268]: 251 (100), 219 (29); MS^3^ [251]: 236 (5), 219 (100), 191 (10)
**18**	28.0	Corytuberine (**18**)	328 [M+H]^+^	MS^2^ [328]: 297 (100), 265 (55); MS^3^ [297]: 265 (100); MS^3^ [265]: 237 (35), 233 (100)
**19**	31.0	Nornuciferine (**19**)	282 [M+H]^+^	MS^2^ [282]: 265 (100), 250 (5); MS^3^ [265]: 250 (100), 234 (21)
**20**	32.1	Anonaine (**20**)	266 [M+H]^+^	MS^2^ [266]: 249 (100); MS^2^ [249]: 219 (100), 191 (30); MS^3^ [219]: 191 (100)
**21**	32.9	Lirinidine (**21**)	282 [M+H]^+^	MS^2^ [282]: 251 (95), 219 (100), 191 (5); MS^2^ [251]: 219 (100), 191 (30); MS^3^ [219]: 191 (100)
**22**	34.5	Nandigerine (**22**)	312 [M+H]^+^	MS^2^ [312]: 295 (100); MS^3^ [295]: 280 (100), 265 (10), 264 (95), 263 (30)
**23**	39.4	Roemerine (**23**)	280 [M+H]^+^	MS^2^ [280]: 249 (100); MS^3^ [249]: 219 (100), 191 (50)

Isolated quaternary alkaloid 6 gave an abnormally weak [M–(CH_3_)_2_NH]^+^ fragment ion in the MS^2^ spectrum, but provided a significant and characteristic [M–C_4_H_9_N]^+^ fragment with *m/z* 285 as the base peak. Furthermore, the [M–C_4_H_9_N]^+^ fragment undergoes subsequent CH_3_OH, CO, OH, and CH_3_ losses. The plausible fragmentation pathway of compound **6** is shown in Scheme 1.

The isomers **7** and **8** were eluted in the same peak at 12.8 min, giving [M+H]^+^ at *m*/*z* 265. They produced [M+H−NH_3_]^+^ at *m*/*z* 248, [M+H−C_4_H_12_N_2_]^+^ at *m*/*z* 177, and [M+H−C_4_H_12_N_2_−CH_3_OH] ^+^ at *m*/*z* 145 in MS^2^. The fragmentation pathways proposed for compounds 7 and 8 are shown in Scheme 1.

## 3. Experimental

### 3.1. General

Optical rotations were measured on a P2000 automatic digital polarimeter. UV spectra were obtained in MeOH on a Jasco V-650 spectrophotometer. IR spectra were recorded in KBr pellets on a Thermo Nicolet 5700 infrared spectrometer. CD spectra were measured on a Jasco J-815 spectropolarimeter. HRESI-MS were obtained on an Agilent 6520 Accurate-Mass Q-TOF LC-MS. NMR spectra were taken on Varian Mercury-400 spectrometer using DSS as references. Strong cation ion exchange resin 001 × 7 was a product of Tianjin Nankai Hecheng S & T Co., Ltd. (Tianjin, China). MCI CHP-20P (Toshiba, 75–150 μm) and RP-C18 (YMC, 40–60 μm) were used for column chromatographic separation. MPLC was performed on an EZ Purifier II flash chromatography system (Shanghai Li Sui E-Tech Co. Ltd. Shanghai, China). Analytical HPLC was conducted on a Waters 2695 pumping system equipped with a Waters 2996 photodiode array detector. The preparative HPLC was performed using a Waters 600 pump, a Waters 2487 detector, and an ODS column (250 mm × 20 mm, 5 μm; YMC). HPLC–MS^n^ analysis was performed with an Agilent G2451AA 6320 Ion Trap LC/MS system. HPLC grade MeCN from Fisher (Fair lawn, NJ, USA) was used for LC-MS/MS analysis. Strata X-C strong cation resin (500 mg/6 mL, Phenomenex, Torrance, CA, USA) was used for SPE. Water was purified with a Milli-Q water purification system (Millipore, Bedford, MA, USA). Ammonium acetate and NH_4_OH were analytical grade.

### 3.2. Plant Material

*M*. *officinalis* was collected from Enshi city, Hubei Province of China, in May 2009, and identified by Prof. Bin Yang. A voucher specimen (NO. 20090518) was deposited at Institute of Chinese Materia Medica, China Academy of Chinese Medical Sciences.

### 3.3. Extraction and Isolation

The dried and powdered stem bark of *M*. *officinalis* (25 kg) was extracted with 70% ethanol (v/w = 7) under reflux for three times. The aqueous alcohol solutions were combined and concentrated *in vacuo* to afford a suspension (5 L). The solution obtained was adjusted to pH 1 using HCl and then partitioned three times with 2.5 L of chloroform. After centrifugation, the supernatant of the H_2_O layer was applied to an ion exchange column (001 × 7; H^+^ form). The column was washed with 50% EtOH (10 L) and then the eluate of 2M NH_4_OH (50% EtOH) was collected. The solvent was evaporated under vacuum to yield the alkaloid fraction (73.6 g). The alkaloid fraction was initially chromatographed on a MCI column, eluted with a gradient of EtOH-H_2_O (0:100 to 95:5), to obtain nine fractions (Fr.1–Fr.9). Fr.2 (15 g, 5% EtOH eluent) was subjected to MPLC over an ODS column, eluted with a MeOH-H_2_O gradient (10:90 to 50:50) to give **10** (3.9 g) and a mixture of **7** and **8** (5 mg). Fr.3 (11 g, 10% EtOH elute) chromatographed on MPLC over an ODS column, eluted with a MeOH-H_2_O gradient (10:90 to 50:50) to give compound **5** (5.0 g) and a mixture (100 mg). This mixture was further separated by preparative HPLC (MeCN:0.1% TFA = 12:88, 10 mL/min) to give compounds **11** (14 mg) and **12** (8 mg). Fr.4 (0.5 g, 15% EtOH eluent) was separated by preparative HPLC (MeOH:0.1% TFA = 35:65, 10 mL/min) to give compound **14** (56 mg). Fr.6 (1.2 g, 25% EtOH elute) was subjected to MPLC with an ODS column, eluted with a MeOH-H_2_O gradient (20:80 to 60:40) to give five subfractions (Fr.6.1–Fr.6.5). Fr.6.3 (40 mg) was separated by preparative HPLC (MeOH:0.1% TFA = 35:65, 10 mL/min) to give compound **6** (10 mg). Fr.7 (1.4 g, 35% EtOH elute) was subjected to MPLC with an ODS column, eluted with a MeOH-H_2_O gradient (20:80 to 60:40) to give six subfractions (Fr.7.1–Fr.7.6). Fr.7.4 (35 mg) was separated by preparative HPLC (MeOH:0.1% TFA = 30:70, 10 mL/min) to give compound **17** (4 mg).

*(S)-4-Keto-magnoflorine* (**6**): White amorphous powder; UV (MeOH) *λ*_max_ (nm): 234, 287, 360; [α]20 D +107 (*c*, 0.11, MeOH); CD Δε (nm): +0.79 (346), –0.29 (297), +0.93 (235) (*c* = 8.45 × 10^−5^ M, MeOH); ^1^H-NMR (D_2_O, 400 MHz) *δ*_H_: 6.87 (1H, s, H-3), 6.55 (1H, d, *J* = 8.0 Hz, H-9), 6.40 (1H, d, *J* = 8.0 Hz, H-8), 3.91 (2H, brs, H-5), 3.75 (3H, s, 10-OMe), 3.70 (3H, s, 2-OMe), 3.31 (3H, s, N-Me a), 2.82 (3H, s, N-Me b), 2.78 (1H, ov, H-7a), 2.16 (1H, dd, *J* = 13.2, 12.8 Hz, H-7b); ^13^C-NMR (D_2_O, 100 MHz) *δ*_C_: 189.4 (C-4), 165.2 (C-1), 154.3 (C-2), 151.5 (C-10), 146.6 (C-11), 134.3 (C-11c), 126.4 (C-7a), 124.1 (C-11b), 122.6 (C-11a), 121.2 (C-8), 116.3 (C-3a), 113.1 (C-9), 107.2 (C-3), 71.4 (C-6a), 69.9 (C-5), 58.1 (10-OMe), 58.0 (2-OMe), 56.2 (N-Me a), 48.0 (N-Me b); ESI-MS *m*/*z*: 356 [M]^+^; HRESI-MS *m*/*z*: 356.1514 [M]^+^ (calcd for C_20_H_22_O_5_N, 356.1498; Δ + 4.64 ppm).

*(R)-3,4-Dehydromagnocurarine* (**11**): White amorphous powder; UV (MeOH) *λ*_max_ (nm): 227, 286; ${\left[\alpha \right]}_{\text{D}}^{20}$–92 (*c*, 0.17, MeOH); CD Δε (nm): +0.54 (297), –0.40 (264), –2.63 (230) (*c* = 6.02 × 10^−4^ M, MeOH); ^1^H-NMR (D_2_O, 400 MHz) *δ*_H_: 7.07 (1H, s, H-5), 6.91 (1H, brs, H-4), 6.71 (2H, d, *J* = 8.8 Hz, H-3', 5'), 6.68 (2H, d, *J* = 8.8 Hz, H-2', 6'), 6.21 (1H, brs, H-3), 5.90 (1H, s, H-8), 4.60 (1H, dd, *J* = 11.2, 4.8 Hz, H-1), 3.87 (3H, s, 6-OMe), 3.52 (3H, s, N-Me a), 3.43 (1H, dd, *J* = 12.4, 4.8 Hz, H-1a), 3.22 (3H, s, N-Me b), 2.92 (1H, dd, *J* = 12.4, 11.2 Hz, H-1a); ^13^C-NMR (D_2_O, 100 MHz) *δ*_C_: 157.4 (C-4'), 150.8 (C-6), 148.9 (C-7), 134.2 (C-2', 6'), 131.0 (C-3), 129.7 (C-1'), 127.6 (C-4), 125.8 (C-8a), 120.8 (C-4a), 119.3 (C-8), 118.0 (C-3', 5'), 114.4 (C-5), 76.2 (C-1), 58.9 (6-OMe), 55.1 (N-Me b), 53.3 (N-Me a), 35.7 (C-1a); ESI-MS *m*/*z*: 312 [M]^+^; HRESI-MS *m*/*z*: 312.1610 [M]^+^ (calcd for C_19_H_22_O_3_N, 312.1599; Δ +3.36 ppm).

*(S)-Magnoflorine* (**5**): ${\left[\alpha \right]}_{\text{D}}^{20}$ +192 (*c*, 0.15, MeOH); CD Δε (nm): +1.92 (317), –12.29 (267), +38.64 (233) (*c* = 3.77 × 10^−5^ M, MeOH) [9].

*(R)-Magnocurarine* (**10**): ${\left[\alpha \right]}_{\text{D}}^{20}$ –62 (*c*, 0.14, MeOH); CD Δε (nm): –1.14 (286), –9.59 (231) (*c* = 1.72 × 10^−4^ M, MeOH).

*(S)-Tembetarine* (**12**): ${\left[\alpha \right]}_{\text{D}}^{20}$ +143 (*c*, 0.11, MeOH); CD Δε (nm): +0.89 (286), +2.80 (237) (*c* = 1.74 × 10^−4^ M, MeOH). 

(*S*)-oblongine (**14**): ${\left[\alpha \right]}_{\text{D}}^{20}$ +103 (*c*, 0.23, MeOH); CD Δε (nm): –0.59 (272), +1.21 (234) (*c* = 1.72 × 10^−4^ M, MeOH).

*(R)-Asimilobine* (**17**): ${\left[\alpha \right]}_{\text{D}}^{20}$ –186 (*c*, 0.25, MeOH); CD Δε (nm): +11.04 (262), –79.13 (233) (*c* = 0.97 × 10^−5^ M, MeOH).

### 3.4. Assessment of Aldose Reductase, Lipase, α-Glucosidase Inhibitory Activities

See [16].

### 3.5. Assessment of DPP–IV Inhibitory Activity

See [17].

### 3.6. Assessment of Cytoxic Activity

See [18]. 

### 3.7. Sample Preparation

Compounds **5–8**, **10–12**, **14**, and **17** were isolated by the authors as described above. Their purities were above 95%, as determined by HPLC analysis.

Powdered stem bark of *M*. *officinalis* (40 mesh, 1.0 g) was extracted with 25 mL 1% HCl in an ultrasonic bath (40 kHz) for 30 min. After cooling, the extracted solution was adjusted to the original weight and then filtered. An aliquot of 5 mL of filtrate was passed through Strata X-C strong cation resin (500 mg/6 mL). Wash the column with H_2_O (5 mL), MeOH (5 mL) and 2M NH_4_OH-MeOH (5 mL) successively. Collected the last elute and dry under the stream of N_2_. The residue resolved in MeOH (2 mL). After filtration with 0.22 μm filter, 10 μL of the aliquot was injected for HPLC–MS^n^ analysis.

### 3.8. Chromatographic and MS Conditions

The sample was separated on a Gemini-NX C18 column (4.6 mm × 250 mm, 5 μm, Phenomenex). The mobile phase was (A) CH_3_CN and (B) 0.1M ammonium acetate (adjust the pH to 7.5 using NH_4_OH). A gradient program was used as follows: 5–30% A at 0–20 min, 30–60% A at 20–35 min, and 60–90% A at 35–40 min. The column temperature was controlled at 35 °C. The flow rate was 1.0 mL/min. 283 nm was chosen as the detection wavelength. The optimized ESI-MS^n^ parameters in the positive ion mode were as follows: skimmer, 40.0 V; capillary exit, 132.3 V; nebulizer, 42.0 psi; dry temperature, 350 °C; dry gas 12.0 L/min. For full-scan MS analysis, the spectra were recorded in the range of *m*/*z* 100–1000. Data-dependent scan program was used in the liquid chromatography/tandem mass spectrometry analysis so that the two most abundant ions in each scan were selected and subjected to MS^n^ (n = 2–3) analyses. The collision-induced dissociation (CID) energy was adjusted to 45%. The isolation width of the precursor ions was 1.0 *m*/*z*.

## 4. Conclusions

In the present study, nine alkaloids including two new compounds, (*S*)-4-keto-magnoflorine (**6**) and (*R*)-3,4-dehydromagnocurarine (**11**), were isolated from the stem bark of *M*. *officinalis*. A comprehensive study of alkaloid profile of *M*. *officinalis* has been conducted. Twenty three alkaloids including 13 aporphines, eight benzylisoquinolines and two amides were detected and tentatively identified by HPLC-MS^n^. Nine major alkaloids were unambiguously identified by comparison with isolated standards. 

## References

[1] Jiangsu New Medical College (1977). Chinese Drug Dictionary.

[2] National Commission of Chinese Pharmacopoeia (2010). Chinese Pharmacopoeia I.

[3] Sarker S.D., Latif Z., Stewart M., Nabar L., Sarker S.D., Maruyama Y. (2002). Phytochemistry of the genus Magnolia. The genusMagnolia.

[4] Cui J.F., Zhang G.D., Song W.Z. (1988). Reversed phase ion-pair HPLC determination of quaternary ammonium alkaloids in the traditional Chinese drug Hou-po (*Magnolia officinalis*). Acta Pharmacol. Sin..

[5] Moriyasu M., Wang J., Zhang H., Lu G.B., Ichimaru M., Kato A. (1996). Isolation of alkaloids from plant materials by combination of ion-pair extraction and preparative ion-pair HPLC using sodium perchlorate. III1) Chinese Magnoliae cortex. Nat. Med..

[6] Guo Z.F., Wang X.B., Luo J.G., Luo J., Wang J.S., Kong L.Y. (2011). A novel aporphine alkaloid from *Magnolia officinalis*. Fitoterapia.

[7] Yu S.X., Yan R.Y., Liang R.X., Wang W., Yang B. (2012). Bioactive polar compounds from stem bark of *Magnolia officinalis*. Fitoterapia.

[8] Ishii H., Imai M., Johji S., Tan S., Chen I.S., Ishikawa T. (1994). Studies on the chemical constituents of *Xanthoxylum nitidum* (Roxb.) D.C. (Fagara nitida Roxb.).II. Examination of the chemical constituents by membrane filtration: Identification of magnoflorine, a water-soluble quaternary aporphine alkaloid. Chem. Pharm. Bull..

[9] Ringdahl B., Chan R.P.K., Craig J.C., Cava M.P., Shamma M. (1981). Circular dichroism of aporphines. J. Nat. Prod..

[10] Matsushige A., Kotake Y., Matsunami K., Otsuka H., Ohta S., Takeda Y. (2012). Annonamine, a new aporphine alkaloid from the leaves of *Annona muricata*. Chem. Pharm. Bull..

[11] Malmberg A. (1984). *N*-Feruloylputrescine in infected *Potato Tubers*. Acta Chem. Scand. Ser. B.

[12] Lu Z.M., Zhang Q.J., Chen R.Y., Yu D.Q. (2011). Study on chemical constituents from branches and leaves of *Polyalthia nemoralis*. Chin. J. Chin. MateriaMed..

[13] Wang E.J., Ma Y.B., Zhang X.M., Jiang Z.Y., Chen J.J. (2008). Five alkaloids from vine stems of *Diploclisia affinis*. Chin. J. Chin. Materia Med..

[14] Liu Q.X., Qiu S.Y., Yu H., Ke Y.X., Jin Y., Liang X.M. (2011). Selective separation of structure-related alkaloids in *Rhizoma coptidis* with “click” binaphthyl stationary phase and their structural elucidation with liquid chromatography-mass spectrometry. Analyst.

[15] Hu Y.M., Su G.H., Sze S.C., Ye W.C., Tong Y. (2010). Quality assessment of *Cortex Phellodendri* by high-performance liquid chromatography coupled with electrospray ionization mass spectrometry. Biomed. Chromatogra..

[16] Li S., Cui B.S., Liu Q., Tang L., Yang Y.C., Jin X.J., Shen Z.F. (2012). New triterpenoids from the leaves of *Cyclocarya paliurus*. Planta Med..

[17] Hu C.X., Huang H., Zhang L., Huang Y., Shen Z.F., Cheng K.D., Du G.H., Zhu P. (2009). A new screening method based on yeast-expressed human dipeptidyl peptidase IV and discovery of novel inhibitors. Biotechnol. Lett..

[18] Zhang P.C., Wang S., Wu Y., Chen R.Y., Yu D.Q. (2001). Five new diprenylated flavonols from the leaves of *Broussonetia kazinoki*. J. Nat. Prod..

